# Top Articles That Are Changing *Neurology: Education* Over the Last Year and Today

**DOI:** 10.1212/NE9.0000000000200125

**Published:** 2024-03-19

**Authors:** Roy E. Strowd

**Affiliations:** From the Department of Neurology, Wake Forest University School of Medicine, Winston Salem, NC.

It has been 2 years since the launch of *Neurology*® *Education* at the 2022 American Academy of Neurology Annual Meeting. Over this period, the journal has grown and expanded considerably. At the conclusion of its second year, there is so much to celebrate and many exciting advances planned in the coming year.

This issue of *Neurology: Education* includes a series of articles that reflect the breadth of topics being discussed in the education literature in clinical neuroscience education today. The Viewpoint article kicks off a perspective on artificial intelligence (AI) in medical education by discussing opportunities and challenges posed by AI in neurologic education.^1^ It is a conversation starter for any educator today. The research articles begin with a study addressing the rising cost of medical education and focuses on neurology-bound trainees. Concerning trends are reported with an increasingly concentrated burden of educational debt for trainees from lower-income families and those identifying as underrepresented in medicine.^2^ This article highlights a significant challenge facing all fields and creating challenges for workforce sufficiency, provider diversity, specialist retention, and a potentially impending policy crisis. Two articles set the stage for future curricular innovations and provide important initial data for completing Kern's 6 steps in curriculum development. Needs assessment data are presented to fill a gap in how residents are trained to care for patients with psychogenic nonepileptic spells (PNESs). This study provides data from a sample of trainees and program directors on preferred methods, timing, and instructional strategies for teaching the care of patients with PNES.^3^ A survey-based study in the United States and Canada addresses training gaps in neurodevelopmental disabilities and provides a roadmap for future curriculum work.^4^

Educators in the clinical neurosciences are not just focusing on the hard skills. A novel patient teach-back method is described in the next article providing hard evidence on how to teach the soft skills in the neurology clerkship. This teach-back approach empowered clerkship students to learn from patients at the bedside and could be a model for others.^5^ Finally, a group of authors describes a novel approach to using morning report to create a question of the week program in 1 neurology training program.^6^ This article melds concepts in microlearning, social learning, and the critical skill of distilling a complex case into a discrete learning *nugget*. There are implications not only for morning report conferences but how distillation of information to generate a testable question can be a teaching tool broadly.

Increasingly, educators are also recognizing the value proposition of teaching for health systems. Educational interventions can be powerful mechanisms to address patient safety and quality of care. This issue's simulation article describes how safety checklist was incorporated into simulation training to improve response times and safety procedures for staff teams in the epilepsy monitoring unit. This is a great example of the power of education to improve healthcare systems and contribute to a learning health system. The journal welcomes more articles of this type.

These articles are just a taste of the breadth and depth of topics covered over the last year in *Neurology: Education*. The top articles from this last year are briefly summarized below.

## Summary of Journal Activity in 2023

Over the last year, the journal has received over 140 submissions spanning all submission types with two-thirds of papers in the education research and curriculum innovation categories and an increasing number of reviews, methods in education research, and viewpoint submissions. The average turnaround for first submission from April to December 2023 was 24 days with an acceptance rate of 26%.

## Must-Read Articles in *Neurology: Education* in 2023

The journal has published some phenomenal articles in the last year including the following *must-read* papers:

### The Inappropriate Consult

Medical consultation has a long-standing history in the practice of medicine.^7^ Whether it is through formal consultation request, curbside consult, e-consult, or some other mechanism, medicine has a culture of seeking input and working together to enhance the care of patients. Nevertheless, there is a pervasive sense that not every consult is needed or appropriate. It takes little time within a resident or physician workroom to hear the grumblings of an inappropriate consult. In this provocatively titled article, the authors deconstruct this concept of an inappropriate consult and describe how discordant expectations from the requesting provider and the specialty consultant leads to a perception of inappropriateness. Both providers have a sense that their own specialized knowledge is common knowledge resulting in cognitive bias. Readers are reminded that consults are for the patient's benefit in situations where the primary provider is unsure, doubtful, or to enhance care.

### Patient-Centered Case Series

Neurologists have always learned from patients and patient cases.^8^ The first forays into active learning were patient rounds and case conferences pioneered by founders like Jean-Martin Charcot and others. A physician would present a patient to an audience of learners and practitioners discussing and demonstrating first-hand the nuances of the neurologic examination, clinical findings that supported a localization, and key features that established a diagnosis. While the coronavirus disease 2019 pandemic and shifts in learning have reduced in many training programs these patient-based teaching conferences, their potential value remains. In this Curriculum Innovation, the authors describe a novel approach to the patient case conference. These educators focused not on diagnostic uncertainty, unique neurologic examinations, and rare zebra presentations, but instead invited patients with conditions that lent themselves to discussion of longitudinal management. Through the story of the illness as told by the patient and neurologist, residents were able to learn the nuances of longitudinal outpatient therapeutic decision-making and be excited by careers in outpatient neurology. In the end, all participants benefited. Patients became closer to their neurologist; neurologists strengthened their relationship with the patient; and residents witnessed positive role modeling and learned outpatient management.

### How to Use Games to Learn

The top review article for 2023 was a review of game-based learning in neuroscience.^9^ This article introduces the concepts of gamification, serious games, and game-based learning. Readers walk through a proposed series of steps that mirror Kern's 6 steps for curriculum development and can be used to create their own educational game.

### Standardizing Fellowship Timelines

The history of neurologic education article takes a look back at the experience of neuromuscular medicine in moving toward a match process.^10^ These authors walk through the history of transitioning to a standardized timeline and 2-way matching system for fellowship applicants. During this 2-year process, challenges were faced and lessons learned in how to establish a universal timeline create clear expectations, enhance diversity and inclusivity, protect applicants and programs, and manage an increase in application review workload. Building from this historical perspective, the authors propose a move toward a unified match system that uses a standardized timeline across all specialties—a herculean effort that would minimize confusion and ensure parity among all applicants.

### Residency *Program* Resiliency

The last must-read article in the Viewpoint category is an interesting perspective on the topic of resiliency.^11^ The authors argue for a systems-based approach to addressing burnout by focusing on resiliency at a residency program level. They discuss how the structure, culture, and framework of a residency program can be used to increase communication, sense of belonging, and shared purpose. As healthcare organizations become increasingly large, establishing programs that reinforce and enhance the resiliency of both the institution and the individuals is vital.

These articles are a high bar for the upcoming year in *Neurology: Education* and provide excellent examples of what the journal is seeking in each of these submission categories.

## A Look Ahead at the Coming Year

The upcoming year will continue to be marked by exciting growth for the journal. In addition to the ongoing Call for Cover Art, several ongoing projects are underway. This includes:

1. New series on medical education research methods: a series on the Fundamentals of Neurology Education Research (the FuNER series) will provide a practical, hands-on approach to conducting high-quality medical education research. How do you use a conceptual framework to guide your education research project? What are qualitative methods and how are they used to conduct an education research study? What are the common quantitative methodologies used in education research? These questions and others will be addressed and applied to specific examples in clinical neuroscience education.
2. Disseminating curricular materials: we are currently working on a mechanism to disseminate peer-reviewed curricular resources. Authors and educators with a new curriculum, course, workshop, seminary series, or other scholarly learning event can share this with the teaching community. These curriculum resource reports will include an executive summary that allows an educator to take the instructor guides, facilitators notes, and resources and implement these directly in their own setting. Stay tuned for additional announcements.

Enjoy this issue of *Neurology: Education*. Happy reading.
